# „Platelet-rich plasma“ (PRP): Analyse der Zusammensetzung bei unterschiedlichem Ernährungsverhalten und Blutentnahmezeitpunkt

**DOI:** 10.1007/s00132-023-04442-x

**Published:** 2023-10-16

**Authors:** Hadrian Platzer, Kristina Dorothea Kubon, Solvig Diederichs, Alena Bork, Simone Gantz, Marcus Schiltenwolf, Tobias Renkawitz, Yannic Bangert

**Affiliations:** Orthopädische Universitätsklinik Heidelberg, Ruprecht-Karls-Universität, Schlierbacher Landstraße 200a, 69118 Heidelberg, Deutschland

**Keywords:** Zirkadiane Rhythmik, Arthrose, Insulin like growth factor I, Interleukin‑6, Thrombozyten, Circadian rhythm, Osteoarthritis, Insulin like growth factor I, Interleukin‑6, Thrombocytes

## Abstract

Die Variabilität von PRP trägt maßgeblich dazu bei, dass bisher noch keine ausreichende Evidenz für einen therapeutischen Einsatz von PRP für muskuloskelettale Erkrankungen besteht. In einer großen Studie untersuchen wir mögliche Einflussfaktoren der PRP-Zusammensetzung. Die hier ausgegliederten Ergebnisse zeigen, dass die Konzentrationen von IL‑6, nicht aber die von IGF‑1 oder der zellulären Bestandteile, im PRP von Veganern gegenüber Omnivoren signifikant und gegenüber Vegetariern tendenziell verringert waren. Dies legt nahe, dass die Ernährung einen bedeutenden Einfluss auf therapeutisch aktive PRP-Bestandteile haben kann. Hingegen schienen die hier untersuchten Komponenten nicht wesentlich durch den Blutentnahmezeitpunkt beeinflusst zu sein. Die Identifikation wesentlicher Einflussgrößen auf die PRP-Zusammensetzung wird essenziell sein, um eine ausreichende medizinische Evidenz für den therapeutischen Effekt von PRP bei orthopädischen Erkrankungsbildern zu generieren.

## Abstract (en)

The variability of PRP is a major contributor to the lack of evidence regarding the therapeutic effect of PRP in musculoskeletal diseases. In a large study, we are currently investigating factors that may influence PRP variability. Interim results showed that concentrations of IL‑6, but not IGF‑1 or cellular constituents, were significantly decreased in PRP samples from vegans compared with omnivores and tended to be decreased compared to samples from vegetarians. This suggests that diet may have a significant influence on therapeutically active PRP constituents. However, the constituents studied here did not appear to be significantly affected by the timing of the sampling. Identification of significant variables affecting PRP composition will be critical to provide sufficient medical evidence for the therapeutic effects of PRP in orthopedic conditions.

**Graphic abstract:**

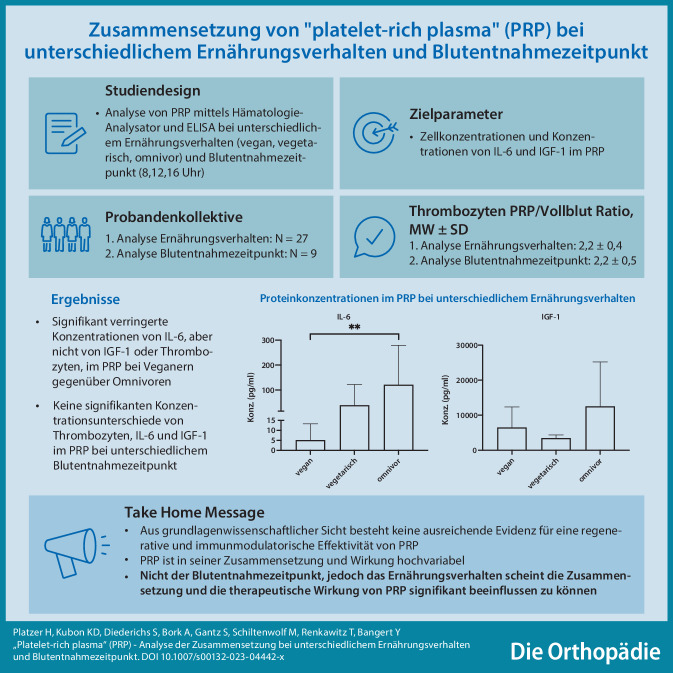

## Einleitung

Autologes thrombozytenreiches Plasma (engl. „platelet-rich plasma“ [PRP]) ist eine an Thrombozyten konzentrierte Fraktion des Vollblutes. Obwohl die PRP-Therapie in der Orthopädie zunehmend an Beliebtheit gewinnt, mangelt es noch an ausreichender Evidenz für einen therapeutischen Effekt von PRP für muskuloskelettale Erkrankungsbilder. Bei einer Expertenbefragung der „Arbeitsgruppe für klinische Geweberegeneration“ der Deutschen Gesellschaft für Orthopädie und Unfallchirurgie (DGOU) beurteilten 89 % der Befragten PRP als nutzbringend, ca. 60 % nutzten PRP in der täglichen Praxis und nur 9 % waren der Auffassung, dass PRP keinen klinischen Nutzen habe [21]. Als häufigste Indikationen in der Orthopädie stellten sich dabei akute und chronische Sehnenpathologien, Muskelverletzungen, Knorpeldefekte und Arthrose heraus. Mit über 2900 auf PubMed gelisteten Veröffentlichungen zu PRP in den letzten 5 Jahren wird belegt, dass die klinische Beliebtheit auch von einer regen Forschungsaktivität begleitet wird.

Ursprünglich wurden Thrombozyten als PRP aufkonzentriert, um als physiologisches Adhäsiv zu dienen [19]. Die zunehmende Erkenntnis über die Komplexität von Thrombozyten, die bei Gefäßverletzung bei weitem nicht nur die Blutgerinnungskaskade in Gang setzen, sondern auf vielfältige Weise aktiv in die Geweberegeneration eingreifen können, weitet jedoch das mögliche Anwendungsspektrum von PRP auf verschiedene regenerative Therapieansätze aus. Thrombozyten können einerseits die in ihren Granula gespeicherten biologisch aktiven Faktoren wie Wachstumsfaktoren und Zytokine ausschütten und andererseits über direkte Zell-Zell-Interaktionen unterschiedliche Gewebe- und Immunzellen beeinflussen [7, 18]. Es wird angenommen, dass Thrombozyten über 300 verschiedene Proteine freisetzen können [9], darunter neben dem typischen Thrombozytenwachstumsfaktor PDGF („platelet-derived growth factor“), den vaskulären endothelialen Wachstumsfaktor VEGF („vascular endothelial growth factor“), den transformierenden Wachstumsfaktor TGF‑β („transforming growth factor“), den insulinähnlichen Wachstumsfaktor IGF‑1 („insulin-like growth factor 1“) und den Hepatozytenwachstumsfaktor HGF („hepatocyte growth factor“) sowie die Interleukine IL‑6 und IL-10. Neben den Thrombozyten als zellulärem Hauptbestandteil finden sich im PRP außerdem auch Leukozyten wie Lymphozyten, Monozyten und Granulozyten, die ebenfalls aktiv in die Geweberegeneration und Inflammation eingreifen können. Diese Vielzahl an potenziell therapeutisch wirksamen autologen Zellen und Zytokinen ist gepaart mit einer simplen Handhabung im klinischen Alltag, und wird unterstützt durch eine Vielzahl kommerzieller Aufbereitungssysteme sowie einer rechtlich einfachen Anwendbarkeit als Arzneimittel.

Die komplexe Zusammensetzung von PRP birgt aber enorme Herausforderungen bezüglich Standardisierung und Vergleichbarkeit von Studienergebnissen. Deren bisheriger Mangel gilt als wesentliche Ursache der unzureichenden Evidenz für eine regenerative und immunmodulatorische Effektivität von PRP in der Therapie orthopädischer Erkrankungsbilder. Die exakte Zusammensetzung des PRP-Therapeutikums aufzuklären ist im Einzelfall zu aufwändig. Variabilität kann jedoch zu einer veränderlichen Wirkung führen, was die Aufdeckung kausaler Wirkmechanismen erschwert, die Vergleichbarkeit unabhängiger Studien empfindlich verringert und so trotz höchsten Evidenzgrades einzelner Studien zu inkonsistenten Ergebnissen führt. Die Aufklärung der Einflussfaktoren auf die PRP-Zusammensetzung ist daher notwendig, um den Evidenznachweis für die Wirkung von PRP bei den zuvor aufgeführten orthopädischen Erkrankungsbildern führen zu können.

Variabilität wird neben standardisierbaren methodischen Unterschieden durch Aufbereitung, antikoagulierender Zusätze und Aktivierungsmethoden der Thrombozyten wahrscheinlich vor allem durch individuelle Unterschiede verursacht. Alter und Geschlecht sind mehrfach als mögliche Einflussgrößen untersucht worden. Übereinstimmend wurden im PRP von jüngeren Individuen signifikant höhere Konzentrationen verschiedener Wachstumsfaktoren wie IGF‑1, PDGF und TGF‑β gemessen als bei älteren [6, 20, 25]. Die Unterschiede der PRP-Zusammensetzung in denselben Studien im Zusammenhang mit dem biologischen Geschlecht sind widersprüchlich, was darauf hindeutet, dass weitere Einflussgrößen von wesentlicher Bedeutung sind.

Hinsichtlich der Zusammensetzung von Vollblut wurde der Einfluss der Diät bereits mehrfach untersucht. In Zusammenhang mit veganer Ernährungsweise wurden geringere Zahlen an Blutzellen, darunter auch Thrombozyten [14, 22], und geringere Mengen zirkulierender Wachstumsfaktoren wie IGF‑1 [1] beschrieben. Untersuchungen inflammatorischer Biomarker, die potenziell von den multiplen pflanzlichen antiinflammatorischen Zytokinen beeinflusst werden können, ergaben laut Metaanalysen geringere Mengen an C‑reaktivem Protein bei Veganern und Vegetariern [10, 16]. Ob solche Unterschiede der Zellzahlen und Proteinkonzentrationen auch ins PRP übertragen werden, ist bisher nicht untersucht worden, kann aber besonders in Bezug auf die regenerative und immunmodulatorische Wirkung von PRP von entscheidender Bedeutung sein.

Es ist anzunehmen, dass die PRP-Zusammensetzung auch intraindividuell variieren kann und hierbei von der allgemeinen körperlichen Verfassung oder dem Immunstatus beeinflusst wird. Dass die Zusammensetzung des Blutes, darunter auch die Zahl und Aktivität von Thrombozyten, im Tagesverlauf schwankt und von Blutkortisonspiegel, zirkadianer Rhythmik und vielen Stressoren beeinflusst werden kann, ist seit langem Gegenstand vieler Untersuchungen [4, 23]. Die Ergebnisse dieser Studien legen nahe, dass hinsichtlich der Zusammensetzung des autologen Blutproduktes PRP ebenfalls Variabilität zu unterschiedlichen Tageszeitpunkten zu erwarten ist. Während in einer frühen Studie periodische Änderungen der Glutathion-Mengen im PRP beschrieben wurden [17], fand eine neuere Studie weder für die Thrombozytenzahl noch für PDGF- und TGF-β-Spiegel tageszeitabhängige Schwankungen [2]. Mit diesen zwei vereinzelten Studien ist offensichtlich die Abhängigkeit der PRP-Zusammensetzung und seiner Aktivität vom Blutentnahmezeitpunkt derzeit erst unzureichend aufgeklärt.

Mit dem übergeordneten Ziel, wichtige individuelle Einflussfaktoren auf die PRP-Zusammensetzung zu identifizieren, führen wir derzeit eine Studie mit einem größeren Probandenkollektiv durch. Hieraus wurden nun die beiden Fragestellungen ausgegliedert, ob die PRP-Zusammensetzung einerseits signifikante Unterschiede zwischen Individuen mit veganer, vegetarischer oder omnivorer Ernährung aufweist und ob das PRP-Profil andererseits wesentlich durch den Blutentnahmezeitpunkt beeinflusst wird, also tageszeitabhängig variiert. Als Hauptevaluationskriterien wurden die Zahl der Thrombozyten und anderer Blutzellen sowie die Spiegel des proinflammatorischen Interleukins IL‑6 und des regenerativen Wachstumsfaktors IGF‑1 erfasst. Die Identifizierung wesentlicher nichtstandardisierbarer Einflussfaktoren der PRP-Zusammensetzung ist von großer Bedeutung, um in Zukunft Patienten- und Probandenkohorten sowie unabhängige Studien besser stratifizieren zu können, somit die Variabilität bei Studienvergleichen zu reduzieren, und so den Evidenznachweis für eine regenerative und immunmodulatorische Wirkung von PRP auf orthopädische Erkrankungsbilder führen zu können.

## Methodik

### Studienkollektiv

Zur Analyse des Einflusses des Blutentnahmezeitpunktes und des Ernährungsverhaltens dienten zwei getrennte Studienkollektive (Tab. 1). Zur Untersuchung des Einflusses des Blutentnahmezeitpunktes auf die Konzentration von IL‑6 und IGF‑1 im PRP wurden neun Probanden, fünf Männer und vier Frauen, mit einem mittleren Lebensalter von 30,4 ± 4,7 Jahren und einem mittleren BMI von 23,0 ± 2,5 kg/m^2^ eingeschlossen. Zur Untersuchung des Einflusses des Ernährungsverhaltens in den letzten 6 Monaten auf die Konzentrationen von IL‑6 und IGF‑1 im PRP wurden 27 Probanden, 14 Männer und 13 Frauen, mit einem mittleren Lebensalter von 25,9 ± 3,7 Jahren und einem mittleren BMI von 22,8 ± 3,6 kg/m^2^ eingeschlossen, neun Probanden für jede untersuchte Ernährungsform: vegan, vegetarisch und omnivor (pflanzliche und tierische Ernährung inklusive Fleisch/Fisch).

**Table 1:** 

	Ernährungsverhalten				Blutentnahmezeitpunkt
	Vegan	Vegetarisch	Omnivor	Gesamt	(8 Uhr/12 Uhr/16 Uhr)
Probanden, *N*	9	9	9	27	9
Geschlecht, N, M/W	5/4	4/5	5/4	14/13	5/4
Lebensalter, MW ± SD (Min-Max)	26,2 ± 4,8 (19–34)	26,2 ± 3,1 (22–30)	25,1 ± 3,2 (20–29)	25,9 ± 3,7 (19–34)	30,4 ± 4,7 (25–37)
BMI (kg/m^2^), MW ± SD (Min-Max)	24,0 ± 5,2 (18,7–33,3)	22,2 ± 3,2 (18,6–26,2)	22,2 ± 1,8 (19,2–25,5)	22,8 ± 3,6 (18,6–33,3)	23,0 ± 2,5 (18,8–27,5)

*BMI* Body-Mass-Index, *MW* Mittelwert, *Min* Minimum, *Max* Maximum, *SD* Standardabweichung, *M* Männlich, *W* Weiblich, *N* Zahl der Probanden

Von dieser Studie ausgeschlossen wurden Probanden mit einer malignen Tumorerkrankung in der Anamnese, einer erfolgten Chemotherapie, einer lokalen oder systemischen Infektions- oder Immunerkrankung, einer Erkrankung des blutbildenden Systems, einer Einnahme von DMARD („disease-modifying anti-rheumatic drugs“) in den letzten 3 Monaten, einer erfolgten Kortisontherapie in den letzten 3 Monaten, einer regelmäßigen Einnahme von NSAR/Paracetamol in den letzten 6 Wochen oder einer Einnahme von Acetylsalicylsäure in den letzten 2 Wochen. Anschließend erfolgte eine ausführliche Anamnese mittels modifiziertem Fragebogen (orientierend an den DEGS-Gesundheits- und Ernährungsfragebögen des Robert Koch-Institutes [Studie zur Gesundheit Erwachsener in Deutschland], dem Fragebogen für den Sportler der Deutschen Gesellschaft für Sportmedizin und Prävention und dem Bewegungs- und Sportaktivitäts-Fragebogen nach Fuchs et al. [8]). Mithilfe des in dieser Studie verwendeten Fragebogens wurde unter anderem das Ernährungsverhalten der Probanden in den letzten 6 Monaten erfasst.

### Probengewinnung, PRP-Herstellung und Probenvorbereitung

Die Blutentnahme erfolgte zum einen in Röhrchen mit EDTA zur Bestimmung der zellulären Vollblutzusammensetzung und zur Gewährleistung eines Ausschlusses von Proben außerhalb des Normbereiches hinsichtlich der zellulären Konzentrationen (Erythrozyten, Thrombozyten, Leukozyten und deren hier nicht dargestellten Subpopulationen Monozyten, Lymphozyten sowie neutrophile, basophile und eosinophile Granulozyten). Zum anderen erfolgte die Blutentnahme mit dem Doppelspritzensystem (ACP-System, Arthrex, Naples, FL, USA) zur Herstellung von PRP, einem autologen konditionierten Plasma (ACP). Zur Analyse des Einflusses des Blutentnahmezeitpunkts auf die PRP-Proteinzusammensetzung erfolgten die Blutentnahmen zu drei verschiedenen klinisch relevanten Tageszeitpunkten (8 Uhr, 12 Uhr und 16 Uhr). Die PRP-Herstellung erfolgte nach Angaben des Herstellers durch Zentrifugation mit 1500 U/min für 5 min (verwendete Zentrifuge: Horizon 24-AH, Drucker Diagnostics, Port Matilda, PA, USA). Im direkten Anschluss an die Blutentnahme und PRP-Herstellung erfolgte die Analyse der zellulären Konzentrationen im Vollblut und PRP mittels automatisiertem Hämatologie-Analysator. Die PRP-Proben wurden zur späteren Proteinanalyse innerhalb von 30 min nach Blutabnahme bei −80 °C tiefgefroren. Zur Freisetzung der thrombozytären Inhaltsstoffe (Aktivierung) wurden zwei Einfrier-Auftau-Zyklen durchgeführt, wobei nach dem ersten Auftauen 20 µl Heparin pro 1000 µl Probe zugefügt wurden, um eine Koagulation zu verhindern.

### Proteinanalyse

Aufgetaute Proben wurden für 10 min bei 1400xg durch eine Filterplatte der Firma Pall Corporation, Port Washington, NY, USA (3 µm „glass fiber“/0,2 µm „supor membrane“) zentrifugiert, um Zellreste der lysierten Thrombozyten vor Analyse zu eliminieren. Im Filtrat wurden die Konzentrationen von IGF‑1 und IL‑6 mittels Enzyme-linked Immunosorbent Assays (ELISA) (R&D Systems, Minneapolis, MN, USA, Lotnummer IGF-1: P310291; Lotnummer IL-6: P320482) nach dem Protokoll des Herstellers ermittelt.

### Statistische Auswertung

Anhand eines Shapiro-Wilk-Test, der Quantil-Quantil-Diagramme und der Fallzahl erfolgte die Beurteilung der Verteilungseigenschaften der Daten. Die statistische Analyse wurde resultierend daraus mit nichtparametrischen Tests durchgeführt. Der Einfluss des Blutentnahmezeitpunktes auf die Konzentrationen von Zellen und IL‑6 und IGF‑1 im PRP wurde mittels Friedmann-Test und bei gegebener Signifikanz mit Wilcoxon-Einzeltests analysiert, der Einfluss des Ernährungsverhaltens erfolgte mittels Kruskal-Wallis-Tests und posthoc Mann-Whitney-Einzeltests. Die Alphafehler-Kumulierung durch multiples Testen wurde durch eine Bonferroni-Korrektur adjustiert. Statistische Signifikanz wurde für *p* < 0,05 angenommen. Alter und Geschlecht waren in den drei Probandengruppen, mit veganer, vegetarischer bzw. omnivorer Ernährung vergleichbar. Signifikante *p*-Werte wurden in den graphischen Darstellungen wie folgt gekennzeichnet: * (*p* < 0,05), ** (*p* < 0,01).

## Ergebnisse

### Zelluläre Konzentrationen im Vollblut und PRP

Die Konzentrationen von Thrombozyten (Normwert: 150–440/nl), Leukozyten (Normwert: 4–10/nl) und Erythrozyten (Normwert: 4–5,2 × 10^3^/nl) im Vollblut lagen für alle eingeschlossenen Probanden im Normalbereich (Tab. 2 und 3). Im Vergleich zum Vollblut war die Thrombozytenkonzentration im PRP bei beiden Studienkollektiven im Mittel 2,2fach erhöht (Kollektiv Blutentnahmezeitpunkt: 568,6 ± 151,5/nl gegenüber 263,3 ± 80,1/nl; Kollektiv Ernährungsverhalten: 523,9 ± 129,9/nl gegenüber 244,5 ± 53,0/nl; Tab. 2 und 3).

**Table 2:** 

		Blutentnahmezeitpunkt			
		08:00 Uhr	12:00 Uhr	16:00 Uhr	Gesamt
*Vollblut, MW* *±* *SD*	Thrombozyten (/nl)	258,7 ± 78,6	270,7 ± 79,1	260,6 ± 91,2	263,3 ± 80,1
	Leukozyten (/nl)	6,1 ± 2,2	7,1 ± 2,1	7,6 ± 2,3	6,9 ± 2,2
	Erythrozyten (x10^3^/nl)	5,0 ± 0,4	4,9 ± 0,4	4,9 ± 0,4	4,9 ± 0,4
*PRP, MW* *±* *SD*	Thrombozyten (/nl)	578,7 ± 170,2	576,7 ± 162,3	550,3 ± 136,8	568,6 ± 151,5
	Leukozyten (/nl)	0,2 ± 0,5	0,1 ± 0,0	0,1 ± 0,1	0,1 ± 0,3
	Erythrozyten (x10^3^/nl)	0,0 ± 0,1	0,0 ± 0,1	0,0 ± 0,1	0,0 ± 0,0
*Ratio Thrombozyten PRP/Vollbut, MW* *±* *SD*		2,3 ± 0,5	2,2 ± 0,4	2,2 ± 0,6	2,2 ± 0,5

*MW* Mittelwert, *SD* Standardabweichung, *nl* Nanoliter, *PRP* „platelet-rich plasma“

**Table 3:** 

		Ernährungsverhalten			
		Vegan	Vegetarisch	Omnivor	Gesamt
*Vollblut, MW* *±* *SD*	Thrombozyten (/nl)	244,0 ± 47,05	249,0 ± 62,4	240,4 ± 54,7	244,5 ± 53,0
	Leukozyten (/nl)	5,3 ± 1,4	7,1 ± 1,9	5,6 ± 1,1	5,0 ± 1,7
	Erythrozyten (x10^3^/nl)	4,7 ± 0,3	4,9 ± 0,4	4,8 ± 0,5	4,8 ± 0,5
*PRP, MW* *±* *SD*	Thrombozyten (/nl)	481,0 ± 119,3	539,2 ± 151,7	551,6 ± 129,9	523,9 ± 129,9
	Leukozyten (/nl)	0,1 ± 0,0	0,1 ± 0,1	0,1 ± 0,1	0,1 ± 0,1
	Erythrozyten (x10^3^/nl)	0,0 ± 0,0	0,0 ± 0,1	0,0 ± 0,1	0,0 ± 0,1
*Ratio Thrombozyten PRP/Vollblut, MW* *±* *SD*		2,0 ± 0,3	2,2 ± 0,4	2,3 ± 0,4	2,2 ± 0,4

*MW* Mittelwert, *SD* Standardabweichung, *nl* Nanoliter, *PRP* „platelet-rich plasma“

Das in dieser Studie verwendete PRP war nahezu vollständig von Leukozyten und Erythrozyten depletiert (Tab. 2 und 3). Es wurden weder für unterschiedliche Blutentnahmezeitpunkte noch für die jeweiligen Ernährungsverhalten der Probanden ein signifikanter Unterschied der Konzentrationen von Thrombozyten im PRP beobachtet.

### Konzentrationen von IL-6 und IGF-1 in Abhängigkeit vom Blutentnahmezeitpunkt

**Figure 1:**
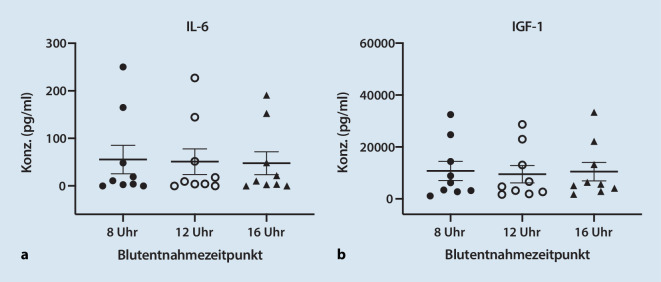

Die Spiegel von IL‑6 und IGF‑1 waren hochvariabel (Abb. 1), zeigten jedoch keine signifikanten Unterschiede zwischen den unterschiedlichen Entnahmezeitpunkten. Die gemessenen IL-6-Werte variierten hierbei von unterhalb der Nachweisgrenze bis 250 pg/ml und die Konzentrationen von IGF‑1 von 1128,7 pg/ml bis 33.342,4 pg/ml. Im PRP von zwei Probanden war die gemessene Konzentration von IL‑6 zu allen Entnahmezeitpunkten gegenüber den übrigen Probanden auffällig erhöht (Abb. 1a). Einer dieser beiden Ausreißer überschnitt sich mit augenscheinlich erhöhten Ausreißern, die es ebenso bezüglich der IGF-1-Spiegel gab. Auch diese waren unabhängig vom Blutentnahmezeitpunkt.

### Konzentrationen von IL-6 und IGF-1 bei unterschiedlichem Ernährungsverhalten

**Figure 2:**
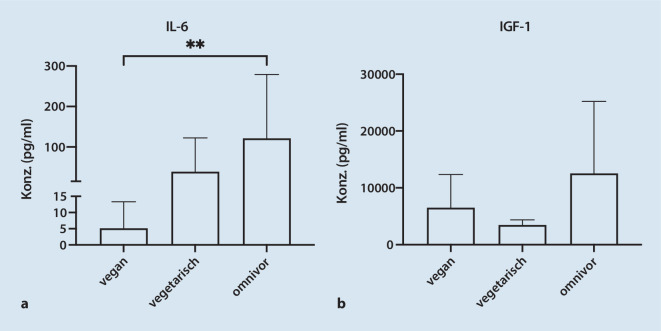

Auch im zweiten Studienkollektiv, das bezüglich der Ernährungsgewohnheiten untersucht wurde, zeigten die Konzentrationen von IL‑6 und IGF‑1 in PRP große interindividuelle Konzentrationsunterschiede (Abb. 2). Es wurden im PRP von Probanden mit omnivorer Ernährungsweise im Vergleich zum PRP von Probanden mit veganer Ernährung eine 24fach höhere mittlere IL-6-Konzentration gemessen (*p* = 0,006). Auch im PRP von Probanden mit vegetarischer Ernährung war die mittlere IL-6-Konzentration gegenüber Veganern um das 8fache erhöht (nicht signifikant, Abb. 2a). Dahingegen zeigte die IGF-1-Konzentration im PRP keine signifikanten Unterschiede zwischen Probanden mit veganer, vegetarischer oder omnivorer Ernährungsform (Abb. 2b).

## Diskussion

„Platelet-rich plasma“ wird regeneratives sowie immunmodulatorisches Potenzial zugeschrieben und es wird in der orthopädischen Medizin zunehmend als Therapeutikum eingesetzt. Ergebnisse experimenteller und klinischer Studien zeigten teils erfolgsversprechende Ergebnisse [3, 11, 15]. Ein eindeutig nachweisbarer medizinischer Nutzen für den Einsatz von PRP in der Orthopädie besteht aufgrund widersprüchlicher Studienlage derzeit nicht. Ein wesentlicher Grund hierfür ist die Variabilität von PRP, deren nichtstandardisierbare Einflussgrößen besser verstanden werden müssen, um zielgerichtete Studien unter Ausschluss von relevanten Störfaktoren durchführen zu können.

Diese Studie soll klinische Parameter identifizieren, die therapeutisch wesentliche Bestandteile von PRP beeinflussen können. Die hier beschriebenen ausgegliederten Untersuchungen zeigten keine konsistenten tageszeitlichen Schwankungen von Zellzahlen oder IL-6- und IGF-1-Konzentrationen im PRP, die jeweils jedoch wie erwartet großen interindividuellen Schwankungen unterlagen. Erstmalig zeigen wir hier, dass die Spiegel des proinflammatorischen IL‑6 im PRP, nicht aber die Spiegel des proliferativen und anabolen Wachstumsfaktors IGF‑1 oder die Konzentrationen einzelner Blutzellen wie Thrombozyten, bei Veganern signifikant niedriger waren als bei Probanden mit omnivorem Ernährungsverhalten und tendenziell niedriger waren als bei Vegetariern. Während dies unseres Wissens nach die erste Studie ist, bei der PRP hinsichtlich eines möglichen Einflusses der Ernährung analysiert wurde, wurde ein solcher Einfluss bereits mehrfach für die Proteinzusammensetzung im Vollblut untersucht. Eine Metaanalyse zeigte, dass die mediterrane Ernährungsweise als Intervention die IL-1β- und IL-6-Spiegel zu senken scheint [13]. IL‑6 kann hochgradig immunmodulatorisch wirken [12] und spielt bei der Pathologie von Sehnenverletzungen und Arthrose eine wichtige Rolle [5, 24]. Auch wenn IL‑6 als klassisches proinflammatorisches Interleukin zählt, ist dessen Wirkweise weit komplexer als ursprünglich angenommen. Durch Induktion verschiedener Signalwege kann es sowohl katabole als auch protektive Prozesse auslösen [24]. Die hier beschriebenen Unterschiede der IL-6-Spiegel im PRP von Probanden mit verschiedenem Ernährungsverhalten legen nahe, dass die Ernährungsweise in Abhängigkeit vom adressierten orthopädischen Erkrankungsbild durch seinen Einfluss auf IL‑6 bei der Therapie mit PRP von Bedeutung sein und die Wirkweise von PRP beeinflussen kann. Weiterführende Studien sind nun nötig, um einen möglichen kausalen Zusammenhang zwischen veganer Ernährung und niedrigem IL-6-Spiegel im PRP zu belegen und zu untersuchen, wie schnell der IL-6-Spiegel auf eine Ernährungsumstellung reagiert.

Die hier dargestellten Ergebnisse der Konzentrationen von IL‑6, IGF‑1 und den zellulären Bestandteilen im PRP legen nahe, dass der Blutentnahmezeitpunkt keinen signifikanten Einfluss auf die PRP-Zusammensetzung hat. Dies steht im Einklang mit Ergebnissen einer Studie von Aoto et al., die keinen Einfluss des Tageszeitpunktes der Blutentnahme bzw. der PRP-Herstellung auf die Konzentration von TGF-β 1, PDGF und der Thrombozytenzahl im PRP fanden [2]. Während dies vielversprechend für die alltägliche praktische Anwendung von PRP ist, ist natürlich nicht ausgeschlossen, dass andere wichtige Komponenten des PRP wie z. B. die Thrombozytenaktivität oder Spiegel anderer Proteine im Tagesverlauf schwanken.

Die hier dargestellten Ergebnisse zeigen bei gleicher methodischer Verarbeitung der Proben große interindividuelle Unterschiede der Konzentrationen von IL‑6 und IGF‑1, sodass davon auszugehen ist, dass hierfür probandenspezifische Faktoren ursächlich sind. Solche Parameter, die als Störfaktoren fungieren könnten, planen wir im Laufe der gesamten Studie – nach Untersuchung weiterer Probandenproben und erfolgter Korrelation der bestimmten Proteinkonzentrationen im PRP mit der Gesamtheit der erhobenen klinischen Daten aus dem verwendeten Fragebogen – zu identifizieren.

Die hier gezeigten Ergebnisse zeigen, wie therapeutisch bedeutende Komponenten wie IL‑6 im PRP bei unterschiedlicher Ernährung schwanken können und tragen somit wesentlich dazu bei, die Variabilität von PRP aufzuzeigen und besser zu verstehen. Diese Ergebnisse basieren auf einer Analyse eines ersten Probandenkollektivs und sind dadurch noch mit Unsicherheiten verbunden, welchen durch den Einschluss weiterer Probanden begegnet werden soll. Weitere Einflussfaktoren und kausale Zusammenhänge zur PRP-Zusammensetzung sind bereits Gegenstand weiterer Untersuchungen, die wir derzeit im Forschungsbereich „molekulare und regenerative Orthopädie“ an der Orthopädischen Universitätsklinik Heidelberg in einem größeren Kollektiv fortführen, damit das Probandenkollektiv erweitert und ergebnisverzerrende Störfaktoren reduziert werden können. Ein belegbarer Nutzen für PRP als Therapeutikum bei unterschiedlichen orthopädischen Erkrankungsbildern ist momentan noch in wissenschaftlicher Diskussion.

## Fazit für die Praxis

- Aktuell besteht aus grundlagenwissenschaftlicher Sicht keine ausreichende Evidenz für die theoretisch formulierte, regenerative und immunmodulatorische Effektivität von PRP bei der Behandlung orthopädischer Erkrankungsbilder.
- „Platelet-rich plasma“ (PRP) ist in seiner Zusammensetzung und Wirkung hochvariabel, was durch die Heterogenität in der Herstellung sowie durch die individuellen patientenspezifischen Merkmale begründet ist.
- Es ist davon auszugehen, dass neben Alter und Geschlecht weitere wesentliche patientenspezifische Einflussfaktoren die PRP-Zusammensetzung bestimmen können, einhergehend mit einer möglichen signifikanten Beeinflussung der Wirkung.
- Die Ergebnisse dieser Studie legen – basierend auf den Messungen zu den zellulären Bestandteilen und den Konzentrationen von Interleukin‑6 und „insulin-like growth factor-1“ im PRP – nahe, dass nicht der Blutentnahmezeitpunkt, jedoch das Ernährungsverhalten einen signifikanten Einfluss auf die Zusammensetzung von PRP und der daraus abgeleiteten therapeutischen Wirkung haben kann.

## Abkürzungen

ACP: Autologes konditioniertes Plasma
BMI: Body-Mass-Index
DEGS: Studie zur Gesundheit Erwachsener in Deutschland
DGOU: Deutschen Gesellschaft für Orthopädie und Unfallchirurgie
DMARD: „Disease-modifying anti-rheumatic drugs“
EDTA: Ethylendiamintetraacetat
ELISA: Enzyme-linked Immunosorbent Assays
HGF: „Hepatocyte growth factor“
IGF: „Insulin-like growth factor“
IGF‑1: „Insulin-like growth factor 1“
IL: Interleukin
MW: Mittelwert
NSAR: Nichtsteroidale Antirheumatika
PDGF: „Platelet-derived growth factor“
PRP: „Platelet-rich plasma“
SD: Standardabweichung
TGF: „Transforming growth factor“
VEGF: „Vascular endothelial growth factor“
